# The validity and reliability of the patient enablement instrument (PEI) after GP appointments in Finnish health care centres

**DOI:** 10.1186/s41687-020-00243-4

**Published:** 2020-09-16

**Authors:** Elina Tolvanen, Tuomas H. Koskela, Mika Helminen, Elise Kosunen

**Affiliations:** 1 Faculty of Medicine and Health Technology, Tampere University, c/o coordinator Leena Kiuru, Arvo Building B, 33014 Tampere, Finland; 2 Pirkkala Municipal Health Centre, Pirkkala, Finland; 3 Research, Development and Innovation Centre, Tampere University Hospital, Tampere, Finland; 4 Faculty of Social Sciences, Health Sciences, Tampere University, Tampere, Finland; 5 Centre for General Practice, Pirkanmaa Hospital District, Tampere, Finland

**Keywords:** Patient enablement instrument, Validity, Reliability, Finland

## Abstract

**Background:**

The aim of this study was to assess the validity and reliability of the Patient Enablement Instrument (PEI) in Finnish health care centre patients. A pilot study was conducted to assess the content validity of the PEI. A questionnaire study in three health care centres in Western Finland was performed in order to assess acceptability, construct validity, internal consistency, and measurement error of the instrument. A telephone interview 2 weeks after the appointment was performed to evaluate reproducibility.

**Results:**

The pilot study with 17 participants indicated good content validity of the PEI. In the questionnaire study, altogether 483 with a completed PEI score were included in the analyses. Factor analysis and item-scale correlations suggested high structural validity. The internal consistency of the instrument was high (Cronbach’s α = 0.93). The PEI score diminished strongly over the two-week period.

**Conclusions:**

The PEI has good content validity and acceptability, good construct validity, high internal consistency but low reproducibility. Thus, the PEI seems to be an applicable tool to measure patient enablement in Finnish primary health care.

## Background

The patient’s experience of care is one of the essential elements when assessing health care quality. To explore this, many health-related patient-reported outcome (HR-PRO) measurements have been created, and new ones are constantly in development [1]. In Finland, the health care system is about to undergo a large reform, and one aspect of this will involve the client’s wider freedom to choose where to obtain health and social services [2]. Under these circumstances, instruments to evaluate health care quality are needed. In addition, in order to evaluate the appropriateness of the available instruments, we need to assess their validity and reliability.

The concepts of validity and reliability are complex and have several definitions and interpretations that are often used interchangeably. The international COSMIN committee has developed a consensus for defining the psychometric properties of HR-PRO measurements [3]. We have used the COSMIN checklist for methodological studies [4] as a guideline when designing the study, as well as the recently published COSMIN Risk for Bias checklist when writing this paper [5].

**Fig. 1:**
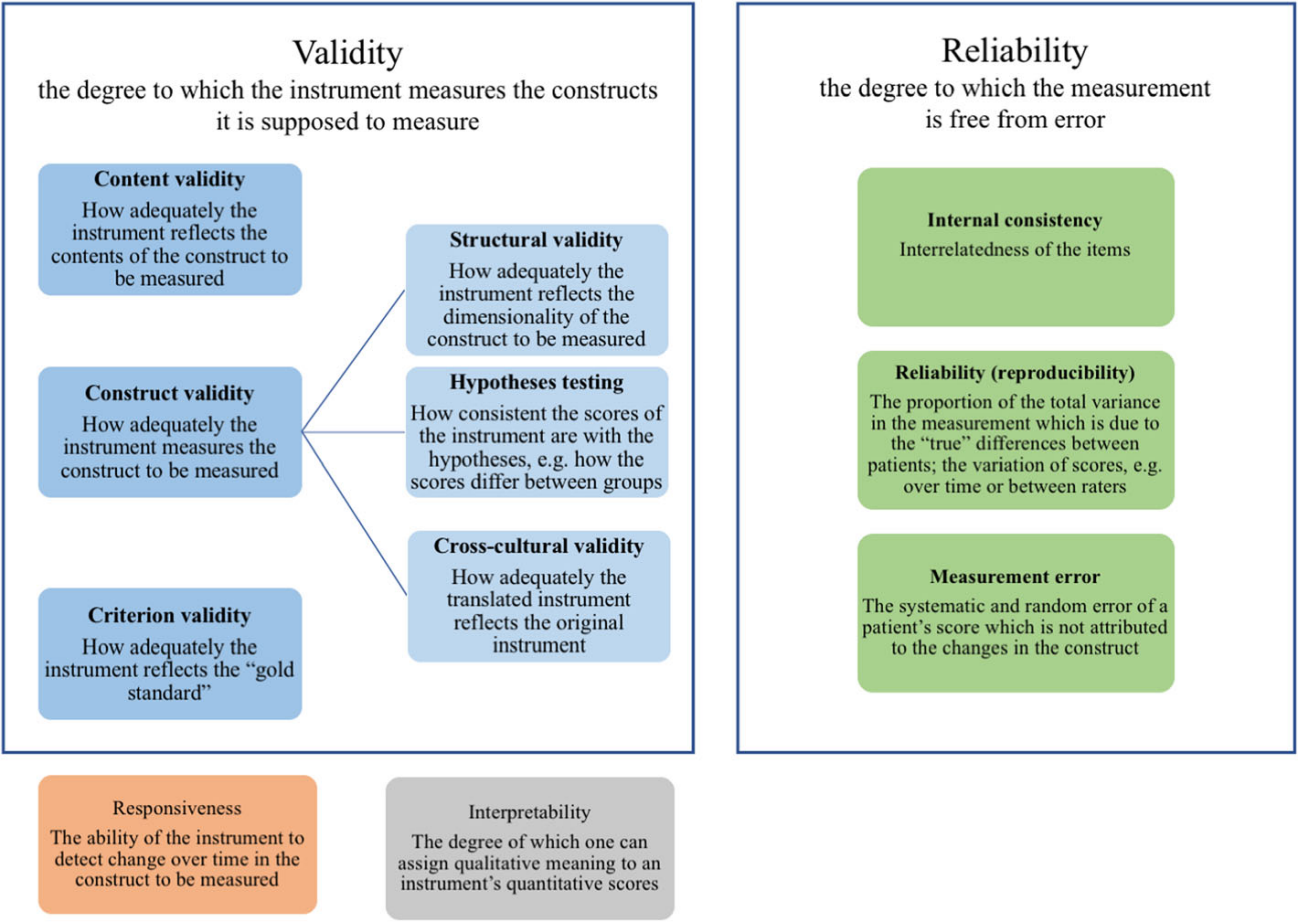
The concept figure of validity and reliability, adapted from: L.B. Mokkink et al. The COSMIN checklist for assessing the methodological quality of studies on measurement properties of health status measurement instruments: An international Delphi study, Qual. Life Res. 19 (2010) 539–549

Figure 1 presents the different domains of validity and reliability that have been adapted from the COSMIN guidelines [4]. According to the COSMIN criteria, the quality of an HR-PRO measurement can be divided into three domains: validity, reliability, and responsiveness [3]. Validity is defined as the degree to which the instrument measures the constructs it is supposed to measure. Reliability refers to the degree to which the measurement is free from measurement error. Responsiveness is defined as the ability of the instrument to detect change over time in the construct to be measured [3]. Furthermore, two separate concepts exist: interpretability refers to the degree to which one can assign qualitative meaning to an instrument’s quantitative scores [3], and acceptability addresses how acceptable the instrument is for the respondents to complete [1].

**Fig. 2:**
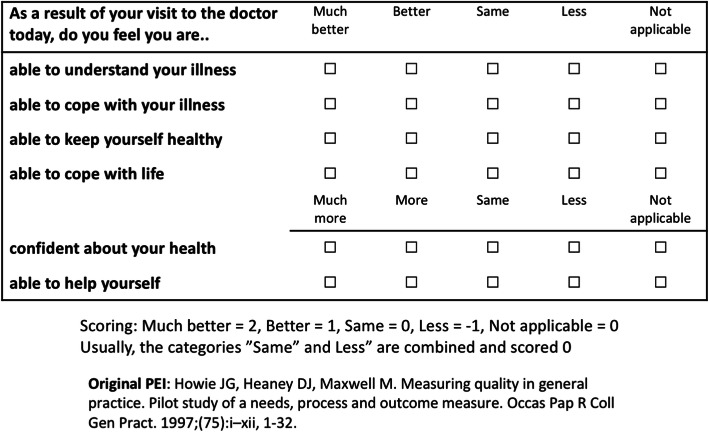
Patient Enablement Instrument

Patient enablement is defined as the patient’s ability to understand and cope with illness and life after a consultation [6]. It is suggested to be a useful HR-PRO in primary health care [6–8]. The Patient Enablement instrument (PEI) is a six-item questionnaire addressed to the patient immediately after a consultation (Fig. 2). The items in the PEI questionnaire enquire the degree to which patients feel able to 1) understand their problem(s)/illness, 2) cope with the problem(s)/illness, 3) keep themselves healthy, 4) cope with life, 5) be confident about their health, and 6) help themselves after a consultation [6]. The PEI has been applied in several countries [6, 9–16].

Regarding factors associated with patient enablement, some studies have found that patient’s older age is associated with higher enablement scores [9, 11, 16]. However, there are contradictory results [10, 13, 14, 17]. Having one [10, 18] or several chronic diseases [19], or lower self-perceived health status [11, 17, 19] have been associated with lower enablement in previous studies. PEI scores also seem to vary according to the patients’ ethnic background [9, 10, 20, 21]. Furthermore, longer consultations, [6, 9, 12, 21–24], positive experiences of doctor–patient communication [10, 25, 26] and the doctor’s empathy [19, 27] have been associated with higher enablement, as has higher patient satisfaction [25, 28]. On the other hand, the PEI seems to measure different outcome compared to patient satisfaction instruments [7, 16, 29, 30].

All items included in the PEI are designed to measure the same underlying concept, namely patient enablement. In earlier studies, the internal consistency of the instrument has been reported to be high [6, 7, 9, 13, 14, 16, 31, 32]. Studies regarding the reproducibility (or reliability over time) of the PEI have produced contradictory results, with either a minimal change over time [14, 33] or lower scores in the retest compared to the baseline [13, 15, 34]. However, there are only a few studies on the PEI in the Nordic countries [13, 35], and none that evaluate the psychometric properties of the PEI in the Finnish context.

The PEI was developed in the UK, where GP consultation times are short (5–8 min) [6, 9, 20, 27] and primary health care is maintained by independent GP practices. In Finland, the universal public health care system is organised by the municipalities, which provide services in multidisciplinary health care centres/stations. The appointments are usually fairly long, from 15 to 30 min, and several issues are usually handled within the same appointment.

The aim of this study is to assess the validity and reliability of the PEI in Finnish health care centre patients, focusing on the acceptability, content and construct validity, internal consistency, and reliability of the instrument.

## Methods

### Study design

**Fig. 3:**
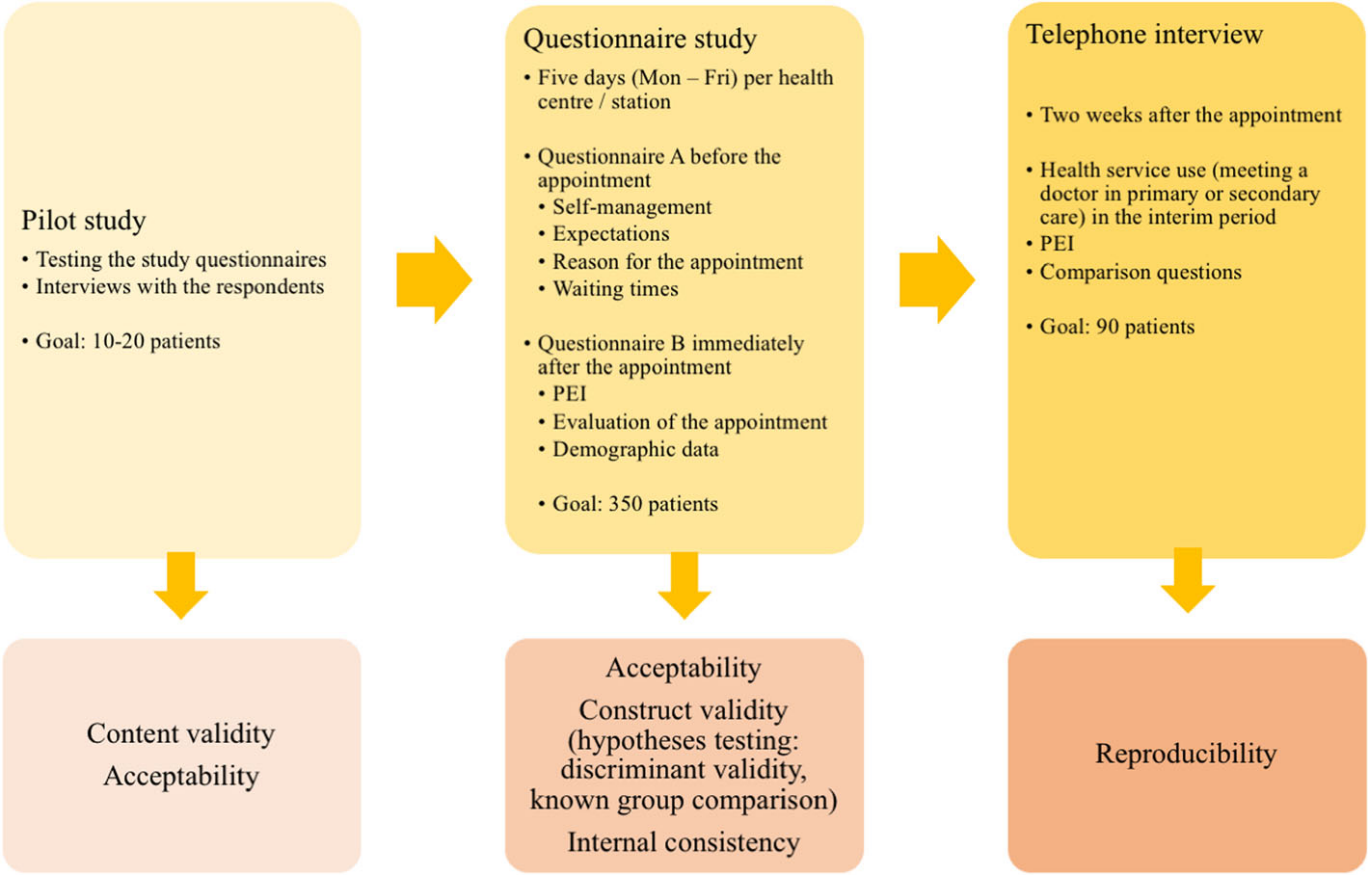
The study design

This study consisted of three parts: 1) a pilot study, 2) a questionnaire study with forms before (A) and immediately after (B) the appointment with a GP at a health care centre, and 3) telephone interviews 2 weeks after the appointment. The study design and the detailed information about the purpose of each part is presented in Fig. 3. In the pilot study, the goal was to recruit 10–20 participants. For an 80% chance of detecting a 0.5-point difference in the PEI score between the two groups, 350 and 90 participants were needed for the questionnaire study and telephone interviews, respectively. Two weeks has been considered a suitable interval for test-retest measurements when evaluating patient-reported outcomes [36]. Furthermore, telephone surveys seem to produce similar results as face-to-face surveys [37].

Questionnaire A (before the appointment) included questions about the patient’s self-management, expectations about the consultation, reason for the appointment, and waiting times. Questionnaire B (after the appointment) included the PEI, other assessments of the appointment, and the patient’s demographic information. The telephone interview included information about health service use in the interim period, the PEI, and comparison questions about patient satisfaction, benefit, involvement, and instruction evaluation. Because the patients should be “stable” between the two measurements (meaning that there had been no new interventions) [4], patients who had visited a doctor in primary or secondary care within the two-week interim period were excluded from the test-retest analyses.

### Patient enablement instrument and item scoring

In 2014, the PEI questionnaire had been formally back-translated into Finnish as a part of a larger study [35]. Our research team, along with one professional translator (naive to both versions of the PEI), evaluated the translation and concluded that it was faithful.

The options in the PEI are “much better/more” (2 points), “better/more” (1 point), “same” (0 points), “less” (− 1 point), and “not applicable” (0 points), thus leading to a sum score ranging from − 6 to 12. Usually, the “same” and “less” options are combined [6, 12, 13], but we wanted to explore whether the negative option should be preserved in the questionnaire, as was done in one previous study [14]. The PEI score could be calculated when at least three of six questions had been answered [6]. Researchers are unanimous on which PEI scores reflect “adequate” or “good” enablement after consultation. For grouping purposes, researchers have used a cut-off point of one (PEI score 0 versus PEI score ≥ 1) [10] or six [6], or have compared PEI scores below and above the average on current study population [19].

The questions which were compared to the PEI indicated patient satisfaction, experienced benefit, patient involvement, and instruction evaluation. The comparison questions are presented in the Table 1. The comparison questions were measured on a 4-point Likert scale.

**Table 1:** The comparison questions

	I fully agree	I partly agree	I partly disagree	I fully disagree	N/A^a^
I would recommend this doctor to a friend or a relative					
I benefited from my appointment with this doctor					
I was involved in the decisions made at the appointment					
I got adequate instructions to carry on with my care					

^a^N/A = not applicable

### Data collection

The study data were collected between February and May 2017. The study was conducted in three municipalities in the Pirkanmaa district in Western Finland: Hämeenkyrö, Pirkkala, and Tampere. Hämeenkyrö is a rather rural county with a sizable elderly population. Pirkkala is a semi-rural county with a relatively youthful population situated next to the large city of Tampere. Tampere is the third largest city in Finland, with 230,000 inhabitants and a sizable population of young adults.

The pilot study was conducted at Pirkkala health care centre in February 2017. During 1 day, the researcher (ET) approached patients in the waiting room of the health centre and asked them to participate. The participants were requested to fill out the study questionnaires and to have a brief interview afterwards with the researcher. The participants had to evaluate e.g. the appropriateness and relevance of the questions.

During the data collection period, the goal was to recruit all patients who had an appointment with a GP at the health centre during a five-day period (Monday to Friday, during office hours). The researcher (ET) or research assistants tried to approach everyone who came to the waiting room of the health centre/station during office hours. All the participants were informed about the study both orally and in writing, and they gave written consent. If the participant had difficulties with filling in the questionnaire (e.g. due to deteriorated vision), the research assistants helped them. The exclusion criteria were age under 18 years, insufficient Finnish skills, and severity of illness preventing participation in the study. In addition, patients who had an appointment with a GP in maternity care or student care were excluded.

### Assessing validity and reliability: data analysis

All the statistical analyses were performed with IBM SPSS version 25.

#### Content validity and acceptability

The content validity of the PEI in the Finnish context was evaluated during the pilot study. In the questionnaire study, all patients who had a valid PEI score after the appointment were included in the analysis. Completion rates, distributions, and the means of the PEI items were analysed in order to assess the acceptability of the instrument.

#### Construct validity

The unidimensionality of the instrument, indicating reliability and structural validity, was evaluated by principal component factor analysis with Varimax rotation. Factor analysis should produce one factor with an eigenvalue > 1, and each component should have similar factor loading. Furthermore, the structural validity was evaluated by item-scale correlations with the hypothesis that they should be higher than 0.7. Hypothesis testing was evaluated by comparing the PEI to questions measuring patient satisfaction, benefit, involvement, and instruction evaluation (indicating discriminant validity), plus known group comparison. The hypotheses were that the correlation between the PEI score and the comparison questions would be less than 0.4; and that the PEI scores would be lower among patients with a non-urgent reason for consultation, more chronic conditions, and a worse state of health; and the PEI is the same across gender and age groups. The Mann–Whitney *U* test and the Kruskal–Wallis test were used to compare distributions across groups.

#### Internal consistency

Internal consistency between the questionnaire items was evaluated by counting the Cronbach alphas with confidence intervals. A value > 0.7 is considered adequate [38].

#### Reliability (reproducibility)

Reliability over time was analysed by kappa statistics. The mean PEI and comparison question scores between the questionnaire study and telephone interview were compared by the Wilcoxon signed rank test.

#### Measurement error

The standard error of measurement (SEM) was calculated with the following formula: $SEM= SD\sqrt{1-r}$, where *SD* is the standard deviation of the test score and *r* is the reliability coefficient of the test, usually Cronbach’s alpha, Cohen’s Kappa, or some similar coefficient [39].

## Results

### Content validity: the pilot study

Altogether, 32 patients heading for a GP appointment were reached, 21 patients gave their consent, and 17 patients completed the pilot study. The mean age of the participants was 59.3 years (range 23–89) and 10 of them (58.8%) were female. In general, the patients accepted the study questionnaires well. The questionnaires were filled thoroughly and the majority of the respondents found the questions important and relevant. After the pilot study, only minor corrections were made to the questionnaires; the PEI part was not changed.

### The questionnaire study

**Fig. 4:**
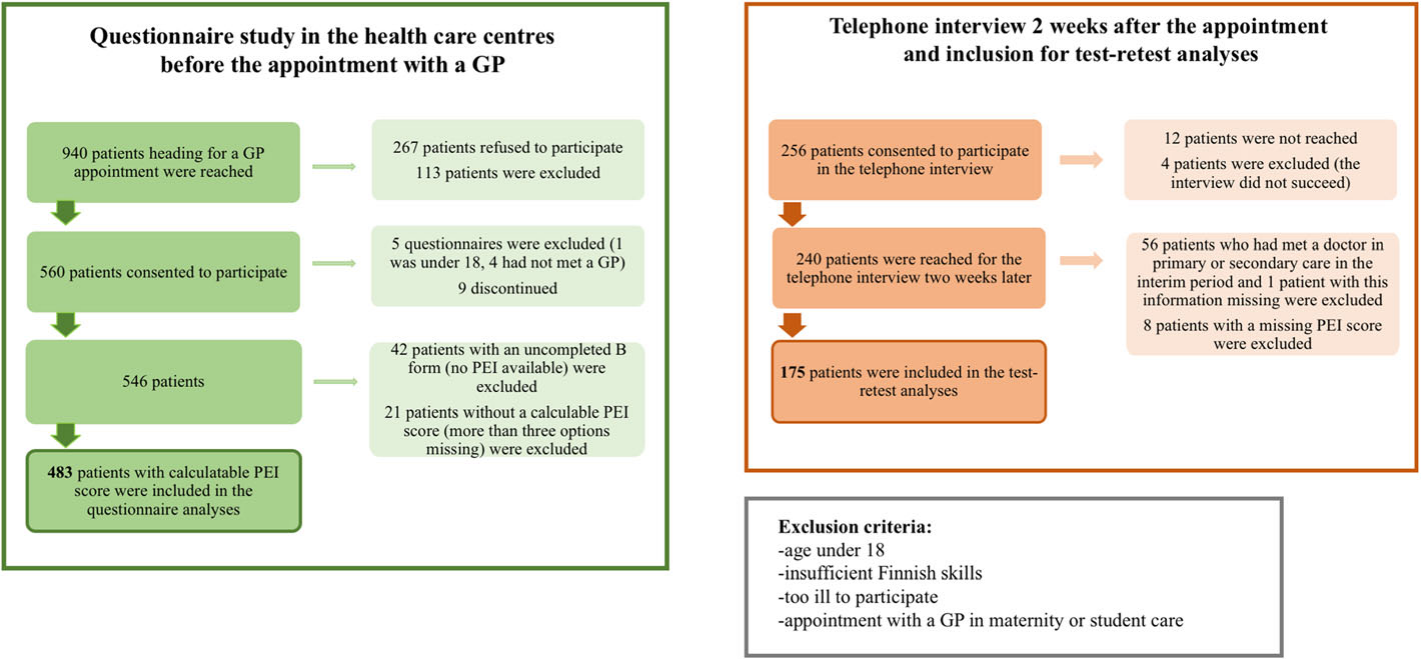
Data collection process and division for the analyses

The data collection in three health centres took a total of 17 days. The patient recruitment process and division for the analyses is presented in Fig. 4. During the data collection period, we reached 940 patients heading for a GP appointment, which was 79.3% of all the patients (information derived from the ICT system in the health care centres). Of those, 546 eligible patients gave their consent to participate. Altogether 118 patients were excluded during the recruitment process, and 63 patients were excluded due to uncompleted questionnaire B or the PEI part.

The demographic factors of the participants are presented in Table 2. Of the 546 participants, 483 patients had a completed PEI score (fewer than three options missing) and were thus included in the analyses. The mean age of the participants was 58.5 years (range 18–97, SD 19.1) and 313 (64.8%) were female. Furthermore, 175 participants were included in the test-retest analyses. When comparing groups by participation in the telephone interview, the groups differed significantly (data not shown). For instance, the telephone interviewees were older and had more chronic illnesses.

**Table 2:** Distributions of the background factors, all participants and by participation in the telephone interview

		All participants, ***n*** = 483		Comparison by participation in the telephone interview			
				Patients who participated in the telephone interview and were included in the test-retest analyses, *n* = 175^a^		Patients who did not participate in the telephone interview, *n* = 254**	
		Frequency	Percentage	Frequency	Percentage	Frequency	Percentage
Age***							
	Range	18–97		**19–88**		**18–97**	
	Mean (SD)	58.5 (19.1)		**62.2 (17.2)**		**56.2 (20.4)**	
	Data missing/NA	17	3.5	7	4.0	9	3.5
**Mean PEI score immediately after the appointment**							
	Mean (SD)	3.78 (3.83)		4.13 (3.95)		3.81 (3.86)	
**Sex**							
	Female	313	64.8	108	61.7	173	68.1
	Male	153	32.8	60	34.3	73	28.7
	Other	1	0.2	0	0	1	0.4
	Data missing/NA	16	3.3	7	4.0	7	2.8
**Language**							
	Finnish	455	94.2	164	93.7	240	94.5
	Other	5	1.1	2	1.1	2	0.8
	Data missing/NA	23	4.8	9	5.1	12	4.7
**Co-habitation**							
	Single, divorced, widowed	199	41.2	72	41.1	105	41.3
	Married, registered partnership, or common-law marriage	267	55.3	96	54.9	140	55.2
	Data missing/NA	17	3.5	7	4.0	9	3.5
Education***							
	No qualifications obtained or primary education (lower-level)	119	24.9	41	23.4	65	25.6
	Upper secondary level of education (middle-level)	245	50.7	**80**	**45.7**	**141**	**55.5**
	Post-secondary or higher (higher-level)	98	20.3	**47**	**26.9**	**37**	**14.6**
	Data missing/NA	21	4.3	7	4.0	11	4.3
Working status***							
	Working	92	19.0	**21**	**12.0**	**61**	**24.0**
	Retired	275	56.9	**112**	**64.0**	**135**	**53.1**
	Other (unemployed, student, other)	99	20.5	**34**	**19.4**	**51**	**20.1**
	Data missing/NA	17	3.5	8	4.6	7	2.8
**State of health (self-assessment)**							
	Excellent	32	6.6	10	5.7	21	8.3
	Good	165	34.2	66	37.5	85	33.5
	Fair	171	35.4	60	34.3	85	33.5
	Poor	18	3.7	6	3.4	7	2.8
	Data missing/NA	97	20.1	33	18.8	56	22.0
Number of chronic illnesses***							
	No chronic illness	78	16.1	**22**	**12.6**	**48**	**18.9**
	One	116	24.0	**38**	**21.7**	**69**	**27.2**
	2–3	191	39.5	**80**	**45.7**	**87**	**34.3**
	More than 3	61	12.6	**26**	**14.9**	**26**	**11.3**
	Data missing/NA	37	7.7	9	5.1	24	9.4
Number of consultation reasons***							
	One	299	61.9	**98**	**56.0**	**170**	**66.9**
	More than one	170	35.2	**71**	**40.6**	**77**	**30.3**
	Data missing/NA	14	2.9	6	3.4	7	2.8
**Consultation reason**							
	Acute	158	32.7	52	29.7	83	32.7
	Non-acute	311	64.4	117	66.9	164	64.6
	Data missing/NA	14	2.9	6	3.4	7	2.8
Location***							
	Semi-rural	147	30.4	**58**	**33.1**	**63**	**24.8**
	Urban	196	40.6	**78**	**44.6**	**108**	**42.5**
	Rural	140	29.0	**39**	**22.3**	**83**	**32.7**

^a^Patients who had not visited a doctor in the interim period and had both PEI scores available

**Patients with no telephone interview and immediate PEI score available

**Statistically significant difference between groups in the Chi-square test (bolded), missing values excluded from the analyses

Previously published in: Tolvanen, E., Koskela, T.H. & Kosunen, E. Comparison of the Patient Enablement Instrument (PEI) with two single-item measures among Finnish Health care centre patients. *BMC Health Serv Res* 19**,** 376 (2019) doi:10.1186/s12913-019-4182-2

### Acceptability

The overall response rate was 64.4% (267 refused + 483 completed). The mean PEI score immediately after the appointment was 3.78 (range 0–12, SD 3.83). Altogether 131 of 483 (27.1%) had the floor (0 points) score and 37 (7.7%) the ceiling (12 points) score. There were only 16 respondents (3.3%) with missing items. In addition, it was not possible to compute the PEI score in 63 of 546 responses (these were excluded from the analyses). Of those, 42 respondents had left the whole of questionnaire B empty, leaving 21 PEI scores (3.8%) that were not calculable.

The distributions of the PEI answers immediately after the appointment are presented in Table 3. The option “less” was chosen 39 times out of 2898 answers (1.3%). In their original work to develop the PEI, Howie et al. decided to merge the “less” and “same” options, because only 1% of respondents chose the option “less” in any of the questions [6]. Thus, we adhered to this conclusion and combined the options “less” and “same”. Furthermore, the option “not applicable” was chosen 86 times out of 2898 answers (3.0%). Altogether 23 answers (0.8%) were missing. In general, the acceptability of the PEI in the Finnish context can be considered good.

**Table 3:** The distributions of PEI answers, n = 483

As a result of your visit to the doctor today, do you feel you are	Much better/ much more, n (%)	Better / more, n (%)	Same or less, n (%)	Not applicable (N/A), n (%)	Missing, n (%)
Able to understand your illness	123 (25.5)	157 (32.5)	191 (39.5)	9 (1.9)	3 (0.6)
Able to cope with your illness	98 (20.3)	138 (28.6)	219 (45.3)	20 (4.1)	8 (1.7)
Able to keep yourself healthy	69 (14.3)	130 (26.9)	260 (53.8)	22 (4.6)	2 (0.4)
Able to cope with life	61 (12.6)	116 (24.0)	289 (59.8)	13 (2.7)	4 (0.8)
Confident about your health	83 (17.2)	141 (29.2)	247 (51.1)	10 (2.1)	2 (0.4)
Able to help yourself	68 (14.1)	138 (28.6)	261 (54.0)	12 (2.5)	4 (0.8)

### Construct validity: structural validity

The unidimensionality of the scale was evaluated by principal component factor analysis with Varimax rotation. The factor analysis produced one factor with an eigenvalue > 1, and it explained 73% of the variance at the baseline and 61% of the variance after the two-week interval. Each scale item had a similar factor loading (data not shown).

Spearman correlations for each item and the PEI score are presented in Table 4. All correlations were strong (Spearman’s rho 0.79–0.84 at the baseline and 0.65–0.76 at the retest) and significant at the 0.01 level.

**Table 4:** Spearman correlations between each item and the PEI score at the baseline and retest

Item	Correlation with total PEI score immediately, n = 483	Correlation with total PEI score 2 weeks after, n = 175
Understand illness	0.82	0.76
Cope with illness	0.84	0.73
Keep yourself healthy	0.82	0.65
Cope with life	0.79	0.67
Be confident about your health	0.83	0.76
Help yourself	0.82	0.76

All correlations were significant at the 0.01 level

### Construct validity: hypotheses testing

The correlations between the PEI items or total PEI score and the comparison questions are presented in Table 5**.** There were weak (Spearman’s rho 0.15–0.33) correlations present.

**Table 5:** Spearman correlations between PEI items or total PEI score and the comparison questions, n = 483

PEI item / Comparison question	I would recommend this doctor to a friend or a relative	I got benefit from my appointment with this doctor	I was involved in the decisions made at the appointment	I got adequate instructions to carry on with my care
Understand illness	0.27	0.28	0.24	0.28
Cope with illness	0.19	0.28	0.24	0.25
Keep yourself healthy	0.15	0.18	0.15	0.22
Cope with life	0.20	0.21	0.19	0.24
Keep confident about your health	0.18	0.27	0.21	0.24
Help yourself	0.26	0.24	0.22	0.24
PEI score immediately	0.32	0.33	0.28	0.33

All correlations were significant at the 0.01 level

The test hypotheses that patients with a worse state of health have lower PEI scores and that there is no difference between groups when considering age and sex were supported (data not shown). There were no differences in the distributions or means of the PEI score when comparing groups by the number of chronic illnesses or the consultation reason (neither acute vs long-term issue nor one vs more than one issue).

### Internal consistency

Cronbach’s alpha for the PEI items was 0.93 (95% CI 0.91–0.94, *p* < 0.001) at the baseline and 0.87 (95% CI 0.84–0.90, *p* < 0.001) at the retest, indicating good internal consistency. It was lower (0.906–0.914 at the baseline and 0.84–0.86 at the retest) when any of the six items were deleted, confirming the interrelatedness of the items.

### Reliability (reproducibility)

When analysing the patients who had participated in the telephone interview and not met a doctor in primary or secondary care in the interim period (*n* = 175), the mean PEI score immediately after the appointment was 4.13 (range 0–12, SD 3.95). After the two-week interval, the mean PEI score was 2.78 (range 0–12, SD 3.0). The Wilcoxon signed rank test showed the difference of means to be statistically significant (Z = -5.29, p < 0.001). Kappa statistics showed only weak agreement (0.23–0.29) on all the questions.

### Reliability (measurement error)

The standard error of measurement for the PEI score was: $SEM=3.83\sqrt{1-0.93}$ = 3.83*0.26 = 0.996 ≈ 1.0 points, using the Cronbach’s alpha coefficient immediately after the appointment. Calculated with the test-retest reliability coefficient (Cohen’s Kappa mean 0.26), the SEM for the PEI in this study was 2.97*0.74 = 2.198 ≈ 2.2 points.

## Discussion

This is the first study to assess the validity and reliability of the Patient Enablement Instrument (PEI) in the Finnish context. The PEI seems to have good acceptability and content validity, good construct validity (a highly unidimensional structure and relatively successful hypothesis testing), high internal consistency, and moderate to low reliability (a moderate standard error of measurement, but a low test-retest reliability) among Finnish health centre patients.

As was the case in this study, the PEI has been well accepted in different languages and countries [8, 11–14, 16]. In this study, the mean PEI score was relatively low (3.78), as in previous studies made in Finland [35], Sweden [13], and the UK (particularly those considering white, English-speaking patients) [6, 9, 10]. The low mean of the PEI score is often due to the relatively high proportion of patients reporting zero points [6, 10, 33]. In earlier studies, the proportion of patients reporting zero points ranges from 5% in Japan [16] to 55% in the Netherlands [33]. In our study, over a quarter (27.1%) of patients reported zero points in the PEI.

The construct validity testing confirmed the unidimensional structure of the instrument, as found earlier [6, 14]. The pre-study hypotheses were partly supported. The PEI had only a weak correlation to questions measuring e.g. patient-perceived benefit or satisfaction, suggesting that these are separate concepts. In addition, PEI scores did not differ across gender and age groups, as in one Swedish study [13]. Against the expectations, the PEI distributions and scores seemed to be very similar regardless the number of chronic illnesses or the reason for the consultation. Although this finding is contradictory to previous studies [17, 19], it might be interpreted that the PEI could be used in heterogenous patient populations.

In this study, the Cronbach’s alpha for the PEI was high (0.93), as in earlier studies [6, 7, 9, 13, 14, 16, 31, 32]. For clinical measurements, alpha > 0.90 is regarded as desirable [40]. On the other hand, high values could reflect overlap or redundancy of the items [41]. Even the use of alpha in general has been questioned [42, 43]. However, the alpha coefficient is only one tool when assessing validity and reliability. In practice, it seems that a three-item version of the PEI [10] or a single question [44] are adequate for measuring patient enablement.

To our knowledge, there are no previous calculations of the standard error of measurement (SEM) for the PEI in the literature. The relatively large SEM is mostly caused by the large variation in scores. This could suggest the heterogeneity and diversity of the feelings of enablement. From one point of view, any increase in the patient’s feelings of ability and coping should be considered a positive feature in itself. On the other hand, it has been suggested that if the patient is active, well-informed, and has good self-management prior to the consultation, even a high quality consultation could lead to “no change”, meaning 0 points in the PEI measurement [45].

The test-retest reliability of the PEI is low, indicating that feelings of enablement seem to diminish after a rather short period of time. This was seen also in previous studies [13, 15, 34]. Nevertheless, it has been suggested that this is not due to the measurement itself, but to a true “dilution” of experience [13, 15]. Furthermore, the scores of the comparison questions also diminished statistically significantly over time (data not shown), a phenomenon found with other HR-PROs previously [34]. This confirms the idea that the overall experience is probably at its highest immediately after the consultation. It is therefore important to get the patient to start the planned intervention immediately after the consultation in order to benefit from the increased feelings of ability and coping.

Originally, Howie et al. developed the PEI as an outcome to study whether it is worth using more time in consultations, which are traditionally short in the UK, usually between 5 and 10 min [6, 46]. In this study, we did not collect information on consultation times, but in the Finnish primary health care system consultations are usually longer, around 15 to 20 min [46], and several issues are taken care of during the same consultation. However, in this study, the mean PEI scores and distributions were very similar to those from the UK [6, 9, 10]. This could indicate that up to a certain point, enablement can be increased by lengthening the consultation time, thus strengthening the patient’s feelings of being listened to and taken care of. Nonetheless, it is possible that when the issues at the consultation multiply and become more complex, enablement is no longer dependent on the consultation duration, but on other features instead.

### Strengths and limitations

Our goal was to reach the total sample of patients who visited a health care centre in 1 week, and we reached the majority of patients heading to GP appointments in the data collection period. Furthermore, the response rate was high. We managed to collect a larger dataset than originally planned, and the statistical power calculation demands were met. The study population matches fairly well the average users of Finnish health care centres, with a slight overrepresentation of female and elderly patients [47]. Regrettably, we could not compare the characteristics of participants and non-participants, and a selection bias is therefore possible. The health care centres were not chosen randomly, but they were located both urban and rural areas with different population structures.

Assessments of the cross-cultural validity, criterion validity, and responsiveness of the PEI were not included in the design of this study. Criterion validity could not be assessed because the PEI itself can be considered the “gold standard” of measuring enablement and there are currently no validated questionnaires on patient enablement in Finnish. In addition, with a cross-sectional study design, the elements of responsiveness could not be evaluated.

Formal research on the validity of the comparison questions has not been made in the Finnish context. Nevertheless, the questions have been used in earlier studies [48, 49]. Indeed, there are very few HR-PRO measurements available that have undergone a strict assessment of their validity and reliability in the Finnish context. With this study, we could assess several aspects of the complex concept of validity and reliability, and this can be considered a major strength.

## Conclusions

The PEI seems to have good psychometric properties among Finnish health centre patients. The results are rather similar to previous studies, even though the Finnish primary care setting is different with e.g. longer consultation times. The strongest features of the PEI are its high internal consistency and structural validity. The low reproducibility of the instrument probably reflects the tendency of feelings of enablement to decrease over time. The elements of responsiveness of the PEI need further evaluation, as do its clinical implications.

Overall, the PEI seems to be an applicable tool for measuring patient enablement – which is considered one aspect of quality – in Finnish health care centres when used immediately after the GP appointment. When assessing quality through the patient’s experience, the PEI could be used e.g. along with patient satisfaction measurements to gain a broader understanding. The PEI is generic and could therefore be suitable for GP patients with heterogenous consultation health issues. To achieve feelings of ability and coping would be important to all patients and thus patient enablement should be promoted in GP appointments.

## Data Availability

The datasets used and analysed during the current study are available from the corresponding author upon reasonable request. After the Patient Enablement in Pirkanmaa study has been completed, the data will be stored to Finnish Social Science Data Archive (FSD).
